# The impact of human adipose tissue-derived stem cells on breast cancer cells: implications for cell-assisted lipotransfers in breast reconstruction

**DOI:** 10.1186/s13287-017-0579-1

**Published:** 2017-05-25

**Authors:** Eva Koellensperger, Lilly-Claire Bonnert, Inka Zoernig, Frederik Marmé, Stefanie Sandmann, Günter Germann, Felix Gramley, Uwe Leimer

**Affiliations:** 1 Clinic for Plastic, Aesthetic and Reconstructive Surgery, Spine, Orthopedic and Hand Surgery - ETHIANUM, Vossstraße 6, 69115 Heidelberg, Germany; 2 Department of Cardiology, University of Frankfurt, Theodor-Stern-Kai 7, 60590 Frankfurt, Germany; 3 Department of Medical Oncology, National Center for Tumor Diseases (NCT) and Heidelberg University Hospital, Im Neuenheimer Feld 460, 69120 Heidelberg, Germany

**Keywords:** Mesenchymal stem cells, Adipose tissue, Breast cancer, Breast reconstruction, Cell-assisted lipotransfer

## Abstract

**Background:**

In this study we evaluated the interactions of human adipose tissue-derived stem cells (ADSCs) and different human breast cancer cell lines (BRCAs) with regard to the safety of cell-assisted lipotransfers for breast reconstruction and a thereby unintended co-localization of ADSCs and BRCAs.

**Methods:**

ADSCs were co-cultured with five different human BRCAs (MCF-7, MDA-MB-231, SK-BR-3, ZR-75-30, and EVSA-T) and primary BRCAs from one patient in a transwell system, and cell-cell-interactions were analyzed by assessing doubling time, migration and invasion, angiogenesis, quantitative real-time polymerase chain reaction (PCR) of more than 300 tumor-associated genes, and multiplex protein assays of 20 chemokines and growth factors and eight matrix metalloproteinases (MMPs). Results of co-culture were compared to those of the respective monoculture.

**Results:**

Quantitative real-time PCR revealed remarkable changes in the expression of multiple tumor-associated genes in co-culture compared to monocultures of both ADSCs and BRCAs. Concomitantly, the concentration of several tumor-associated proteins, such as cytokines and MMPs, were strongly increased in co-culture. Furthermore, exclusively in co-culture with ADSCs, the different BRCAs were exposed to several important tumor-modulating proteins, such as CCL2, HGF, or interleukins.

Co-culture did not significantly affect cellular proliferation of either ADSCs or BRCAs (*p* > 0.05). The migration of MCF-7 and MDA-MB-231 BRCAs was significantly increased in co-culture with ADSCs by a mean of 11% and 23%, respectively (*p* = 0.04 and 0.012), as well as that of ADSCs in co-culture with MDA-MB-231, ZR-75-30, and EVSA-T (+11–15%, *p* = 0.035–0.045). Co-culture with MDA-MB-231, SK-BR-3, and EVSA-T BRCAs significantly increased the invasive behavior of ADSCs by a mean of 24–41% (*p* = 0.014–0.039). There were no significant differences in the in vitro invasive properties of BRCAs in co-culture compared to monoculture. An in vitro angiogenesis assay revealed an increased tube formation of conditioned media from co-cultured BRCAs and ADSCs compared to the respective monocultures.

**Conclusion:**

This study further elucidates the possible interactions of primary human ADSCs with human BRCAs, pointing towards a potential increased oncological risk which should not be neglected when considering a clinical use of cell-assisted lipoaspirates in breast reconstruction.

**Electronic supplementary material:**

The online version of this article (doi:10.1186/s13287-017-0579-1) contains supplementary material, which is available to authorized users.

## Background

Adipose tissue has been used for tissue augmentation for a long time, with Neuber being the first to describe such a procedure in 1893. Ever since, surgeons and scientist have tried both to optimize the clinical outcome and to understand the basic science behind the survival or failure of different fat grafts. Over time, the importance of the included undifferentiated progenitor cells, e.g., adipose tissue-derived mesenchymal stem cells (ADSCs), has been emphasized.

ADSCs have been shown to secrete various growth factors transmitting tremendous effects on vascular growth, cell migration and differentiation, and presumably fat graft survival, through a paracrine fashion. Due to that, an increase in ADSC number per volume (cell-assisted lipotransfer (CAL)) has been thought to be beneficial to fat graft survival and overall clinical outcome of lipotransfer [1–3]. In general, adipose tissue transplantation is regarded as a safe and advantageous procedure for breast augmentation or re-shaping in different clinical situations [4]. Based on this, more and more lipotransfers are performed in women with a history of breast cancer, either as a sole procedure or in combination with, for example, implant devices. With regard to the paracrine activity of ADSCs, the question arises whether there is an interaction of ADSCs with potentially remaining local tumor cells in the breast and how that might affect the clinical outcome. Overall, so far, the use of “regular” fat transplants without an additional increase in stem cell number has not shown a significantly increased risk of invasive breast cancer recurrence or a reduced outcome compared to alternative treatments [4]. This, however, might not be true for CALs with an increased number of ADSCs. When aiming for an increased number of stem cells per volume, such as with a CAL, the suctioned fat is further processed, mainly by centrifugation or enzymatic digestion. With this, stromal cells, such as stem cells, are dislocated from their former natural niche and separated from their main cell-cell contacts. This microenvironment, however, is of crucial importance for the fate and behavior of mesenchymal stem cells [5]. By adding isolated cells to a native fat sample, the added stromal cells initially are not anchored in a controlling niche and are thus prone to migration, e.g., towards chemotactic signals of adjacent tumor cells, or changes in proliferation, gene, or protein expression.

It has already been shown that the microenvironment of breast cancers, which is crucial for tumor progression, contains recruited mesenchymal stem cells, also from adipose tissue [6, 7]. The surrounding adipose tissue with its ADSC-containing stromal vascular fraction is supposed to especially contribute to the vascular and fibrovascular stroma of adjacent breast tumors [8]. BRCAs can stimulate the secretion of the chemokine (C-C motif) ligand 5 (CCL5) from bone marrow-derived mesenchymal stem cells (BMSCs), which then act in a paracrine fashion on the BRCAs enhancing their motility, invasion, and metastasis [9]. Furthermore, Jotzu et al. have shown that breast cancer cells are able to induce a change in ADSC morphology and function to a myofibroblast-like cell type, similar to cancer-associated fibroblasts (CAFs) [10]. CAFs have been suggested to enhance tumor growth by secreting stromal-derived factor-1, which directly stimulates cancer cell growth and motility, to support angiogenesis by recruiting endothelial precursor cells into the tumor microenvironment, and to promote epithelial-to-mesenchymal transition (EMT) of tumor cells, a key event in cancer progression [6].

To gain further insight into the respective interactions, we co-cultured primary human ADSCs with five different BRCAs cell lines and primary BRCAs (pBRCAs). We analyzed the proteins secreted in their shared media, quantified the changes in proliferation, migration, invasion, angiogenesis, and gene expression of both cell types, and compared each of the results to that of the respective monocultures of the same cell type.

## Methods

All chemicals, if not noted separately, were purchased from Sigma-Aldrich, Munich, Germany.

### Donor specification

This study was conducted under the guidelines and with the approval of the ethical committees of the University of Heidelberg and of the medical association of the local district Baden-Wuerttemberg, Germany (Reference numbers S-462/2010 and S-022/2013). After informed consent, freshly excised subcutaneous adipose tissue of three men and three women with an age range of 18–42 years (median age 27.5 years) undergoing elective plastic surgery was used for isolation of ADSCs.

Primary BRCAs were isolated from ascites aspirate of a female breast cancer patient (58 years old) with metastasized bilateral invasive lobular breast cancer.

### ADSC isolation and culture

#### Isolation of ADSCs

ADSCs were isolated from freshly excised subcutaneous adipose tissue or liposuction using a procedure modified from Hauner et al. [11]. In brief, the adipose tissue was washed in 1% bovine serum albumin (BSA)/phosphate-buffered saline (PBS), minced, and digested enzymatically by collagenase (collagenase CLS, 220 U/mg; Biochrom AG, Berlin, Germany; 1.5 mg/ml in 1% BSA/Krebs-Ringer solution) for 45 min under constant shaking at 37 °C. Mature adipocytes and connective tissue was separated by centrifugation (700 × g, 7 min, at room temperature). The sedimented cells were resuspended, passed through a 100-μm mesh filter (Neolab, Heidelberg, Germany) and washed twice with 1% BSA/PBS. After erythrocyte lysis (3 min, 155 mM ammonium chloride, 10 mM potassium bicarbonate, 0.1 mM EDTA) cells were washed again twice and plated at a density of 2 × 10^4^ cells/cm^2^ in an expansion medium (see below). After 24 h the medium was changed to remove nonadhered cells.

#### Expansion of ADSCs

ADSC were cultivated in an expansion medium consisting of 60% Dulbecco’s modified Eagle’s medium (DMEM) low glucose (1 g/l d-glucose) (Invitrogen, Life Technologies, Darmstadt, Germany), 40% MCDB-201, 1 × insulin, transferrin, selenous acid (ITS; Becton Dickinson), 10^–8^ M dexamethasone, 0.1 mM ascorbic acid-2-phosphate, 2% fetal calf serum (FCS; Biochrom), 100 U/ml penicillin (Biochrom), 0.1 mg/ml streptomycin (Biochrom), 10 ng/ml recombinant human epidermal growth factor (EGF), and 10 ng/ml recombinant human platelet-derived growth factor-BB (rhPDGF-BB; CellSystems, Troisdorf, Germany). The medium was changed every other day. Once the cells reached 70% confluence they were detached with 0.25% trypsin-EDTA (Biochrom) and replated with 3.5 × 10^3^ cells per cm^2^. ADSCs were incubated at 37 °C with 5% CO_2_ and cultured to passage four.

Passage four cells of all six donors were mixed equally and used as a pool for the following experiments.

### Determination of ADSC stemness

#### Adipogenic differentiation and Oil Red Staining

Adipogenic differentiation was induced as described previously [12]. In brief, ADSCs were seeded in expansion medium at a density of 24,000 cells/cm^2^. After reaching 90% confluence adipogenesis was induced by the alternated use of basal medium (10% FCS/DMEM) supplemented with IDI-mix (500 μM 3-isobutyl-1-methylxanthine, 1 μM dexamethasone, 1 μM indomethacin) for 2 days followed by basal medium plus 10 μg/ml insulin for 1 day. The induction cycle was repeated three times. To confirm the successful adipogenic differentiation, cytoplasmic triglyceride lipid droplets were stained with the Oil Red O staining method as described previously [13].

#### Osteogenic differentiation and Alizarin Red Staining

Osteogenic differentiation was induced as described previously [12]. After seeding at a density of 24,000 ADSC/cm^2^, cells were grown in expansion medium to 90% confluence. Osteogenic induction was initiated by changing the medium to DMEM containing 10% FCS, supplemented with 50 μM l-ascorbate-2-phosphate, 0.1 μM dexamethasone, and 10 mM β-glycerophosphate disodium salt. On day 28 calcium deposition was demonstrated histochemically by Alizarin red staining as follows: monolayers of mineralized mesenchymal stem cells were washed twice with excess PBS and fixed with pre-chilled 70% ethanol for 1 h at –20 °C. After a short washing step with H_2_O the cell layer was incubated with 40 mM Alizarin red (pH 4.2) for 5 min at room temperature. After aspiration of unincorporated dye, cells were washed twice with H_2_O and once with PBS before microscopic analysis.

#### Flow cytometry

ADSCs of all donors were expanded separately to passage four, pooled, and examined once for surface marker expression using flow cytometry as a pool of six donors. The following monoclonal antibodies conjugated to fluorochromes were used: anti-CD11b-APC, anti-CD13-APC, anti-CD29-PE, anti-CD31-FITC, anti-CD34-FITC, anti-CD44-APC, anti-CD45-FITC, anti-CD63-FITC, anti-CD73-PE, anti-CD90-APC, anti-CD105-FITC, anti-CD106-APC, anti-CD-166-PE, and anti-CD235a (all from Becton Dickinson, Heidelberg, Germany). Isotype antibodies were included for all fluorochromes.

Cells were detached with 0.25% trypsin-EDTA, washed in FACS buffer (1% FCS, 0.1% NaN_3_ in PBS), incubated with directly conjugated monoclonal antibodies (5 μl/100,000 cells) in FACS buffer for 30 min on ice, washed twice with FACS buffer, and fixed with 1% paraformaldehyde/PBS. Cells were analyzed using a FACSCanto flow cytometry system (Becton Dickinson). Data acquisition was performed with Diva software (Becton Dickinson) and data were analyzed using FCS express V3 (De Novo Software).

### ADSC-BRCA co-culture

BRCA cell lines MDA-MB-231, SK-BR-3, MCF7, and ZR75-30 were purchased from American Type Culture Collection (ATCC), Manassas, USA (Catalog No. CRM-HTB-26, HTB-30, HTB-22, and CRL-1504), and EVSA-T was purchased from Leibniz-Institute DSMZ GmbH, Braunschweig, Germany (Cat. No. ACC-433).

After informed consent, primary BRCAs were isolated from ascites aspirate of a female breast cancer patient with metastasized bilateral invasive lobular breast cancer. In brief, the ascites aspirate was centrifuged at 490 × g for 10 min and the aspirate supernatant was collected for preparation of conditioned medium consisting of 40% aspirate plus 60% RPMI-1640. The cell pellets were washed in RPMI without supplements and plated on tissue culture plates in RPMI. After an incubation time of 1 h at 37 °C and 5% CO_2_ macrophage adherence occurred, the supernatant containing the nonadherent cells (tumor cells included) was centrifuged and plated on new tissue culture dishes in conditioned medium at a density of 1 × 10^5^ cells/cm^2^. The cells which were adherent after 3 days of culture were expanded to passage four and applied to the co-culture system as primary BRCA cells. The cells were initially analyzed for their cell surface prolife in flow cytometry and were EpCAM-positive, HER2-positive, and negative for estrogen and progesterone receptors (data not shown).

Co-culture of tumor cells and ADSCs was performed in a transwell system. Either 2 × 10^4^ ADSCs or BRCA cell lines (2 × 10^4^ of MDA-MB-231, 4 × 10^4^ of SK-BR-3, MCF-7, and EVSA-T, or 1 × 10^5^ of ZR75-30, with a preculturing of 3 days for SK-BR-3, MCF-7, and ZR75-30), respectively 3 × 10^4^ ADSCs or 1.4 × 10^5^ primary BRCAs, were seeded onto in a polyester membrane transwell-clear insert (Corning; pore size 0.4 μm) while the corresponding other cell type was seeded onto the bottom of a six-well cell culture plate at the cell density described above.

Cells were cultured for up to 5 days in 4 ml ADSC expansion medium (for co-cultures with MDA-MB-231, SK-BR-3, MCF-7, EVSA-T, ZR75-30) or a medium containing DMEM low glucose (1 g/l d-glucose) (Invitrogen, Life Technologies, Darmstadt, Germany) supplemented with 10% FCS (Biochrom), 10 mM Hepes (Gibco, Life Technologies, Darmstadt, Germany), 1% nonessential amino acids (Gibco, Life Technologies, Darmstadt, Germany), 100 U/ml penicillin (Biochrom), and 0.1 mg/ml streptomycin (Biochrom) for co-cultures with primary BRCAs without medium change. Each day cell culture supernatants were harvested and the cell number was determined after trypzinization and trypan blue staining. BRCAs as well as ADSCs alone (either in transwell inserts or on six-well culture plates) served as control and were treated as for the co-culture. For further analysis, the exponential growth phase of the cells was determined and the supernatants of day 4 (MDA-MB231, SK-BR-3, MCF-7, and EVSA-T) or day 5 (ZR-5-30, primary BRCAs) were analyzed in a protein assay while the corresponding cells were used for gene expression studies.

### Determination of cell proliferation

In order to obtain separate growth kinetics during the exponential growth phase for both separate and co-cultured cells, cells of six wells per condition (ADSCs alone, BRCAs alone, and both cell types in co-culture) were harvested with trypsin/EDTA once every 24 h from day 1 to day 5. The cells were stained with trypan blue and the viable cells were counted with a Neubauer chamber. The generation time was calculated by the formula: G (in hours) = (log2 × *T*)/(log*Y* − log*X*), with *T* = time in culture (in hours), *Y* = number of cells at the end of *T*, and *X* = number of cells at the beginning of *T*. The mean generation time was calculated for each condition and the results were evaluated using student’s *t* test.

### Analysis of cell migration

In order to determine the migration capacity of ADSCs and BRCAs alone and in co-culture, the QCM 24-Well Colorimetric Cell Migration Assay (Merck Millipore # ECM 508) was performed. For this purpose, cells of each type were seeded in expansion medium either on the bottom of the supplied 24-well plate (6000 cells per well) or onto the membrane of the transwell insert (6000 cells per insert). Nine wells per condition were seeded and analyzed. Cells were cultured separately for 24 h before co-culture conditions (ADSCs on the well plate bottom, BRCAs in the transwell inserts and vice versa) were established for a further 24 h. Both cell types alone in the inserts without the respective second cell type on the bottom plate served as controls. For evaluation of the assay, the medium was removed and the inserts transferred into new wells containing 400 μl cell stain for 20 min. The inserts were washed with water and the nonmigrated cells were removed from the interior of the inserts with cotton-tipped swabs. The dried inserts were transferred into 200 μl of Extraction Buffer for 15 min and the optical density of 100 μl extracted dye was measured at 560 nm. The results were evaluated using student’s *t* test.

### In vitro analysis of invasive behavior

The invasion capacity of ADSCs and BRCAs was tested in a Cell Invasion Assay Kit (QCM ECMatrix Cell Invasion Assay, Merck Millipore # ECM 550). Cells of each type were seeded in expansion medium either on the bottom of the supplied 24-well plate (6000 cells per well) or onto the membrane of the transwell insert (6000 cells per insert). Nine wells per condition were seeded and analyzed. Cells were cultured separately for 24 h before co-culture (ADSCs on the bottom and BRCAs in the inserts and vice versa) was induced for a further 72 h. Both cell types alone in the inserts without the respective second cell type on the bottom plate served as controls. Next, the medium was removed, the noninvading cells of the interior of the inserts were cleared with cotton-tipped swabs, and the inserts transferred into 500 μl of staining solution for 20 min. Inserts were washed with water, air-dried, and transferred into 200 μl of extraction buffer. The optical density of 100 μl extracted dye was measured at 560 nm. The results were evaluated using student’s *t* test.

### Quantitative real-time polymerase chain reaction (PCR)

The analysis of gene expression was carried out for 261 different genes in three main tumor associated areas: chemokines, cancer regulation by Stathmin1, and metastasis. Real-time PCR was performed at the end of the exponential growth phase of the respective cell cultures. This was identified by daily counting of cell numbers in parallel sets of equivalent cell cultures. This exponential growth phase ended at day 5 for ZR-75-30 and pBRCAs, and at day 4 for all other cell types.

Total RNA was isolated from ADSCs and BRCAs, either cultured alone or in co-culture for 4 days (MDA-MB-231, SK-BR-3, MCF7, and EVSA-T) or 5 days (ZR75-30, primary BRCAs), using the Trizol plus Kit (Life Technologies, Carlsbad, USA). Cells from six separate culture wells per condition were analyzed. The RNA concentration was calculated by Quant-iT RNA-Assay (Life Technologies) and 1 μg was subjected to cDNA synthesis by the High Capacity cDNA Reverse Transcription Kit (Life Technologies). Gene expression analysis was performed on a Step One Plus Instrument (Life Technologies) using TaqMan Real Time PCR technology. Gene expression was analyzed using predesigned TaqMan 96-well array plates each containing 92 different genes of interest and four endogenous controls with 10 ng cDNA per well (Human Chemokines #4418729, Human Tumor Metastasis #4418743, Human Breast Cancer Regulation by Stahmin1 #4418757; Life Technologies, Carlsbad, USA). In order to further investigate a potential EMT of the cells during co-culture the gene expression of E- and N-cadherin was analyzed using specific TaqMan gene expression assays (Hs01023894 for E-cadherin, Hs00983056 for N-cadherin) with 17 ng of cDNA per sample. In addition, a potential receptor modulation of estrogen receptor, progesterone receptor, and ErbB2 was investigated by analyzing 17 ng of cDNA per sample with appropriate TaqMan gene expression assays (Hs00174860 for ESR1, Hs01100353 for ESR2, Hs01556702 for PGR, Hs01001580 for ERBB2; Life Technologies, Carlsbad, USA).

Calculating the difference between the cycle threshold (CT) of the genes of interest and the CT of the endogenous controls from the same sample provided delta-CT values. The PCR analysis was performed three times with equal samples.

### Human cytokine magnetic 30-plex panel

In order to quantify the level of 30 cytokines (CCL2, CCL3, CCL4, CCL5, CXCL-9, CXCL-10, EGF, Eotaxin, fibroblast growth factor (FGF)-2, granulocyte colony-stimulating factor (G-CSF), granulocyte-macrophage colony-stimulating factor (GM-CSF), hepatocyte growth factor (HGF), interferon (IFN)-α, interleukin (IL)-1β, IFN-γ, IL-1ra, IL-2, IL-2r, IL-4, IL-5, IL-6, IL-7, IL-8, IL-10, IL-12, IL-13, IL-15, IL-17, tumor necrosis factor (TNF)-α, and vascular endothelial growth factor (VEGF)), seven different matrix metalloproteinases (MMP1, 2, 3, 7, 8, 9, 10) as well as the extracellular matrix metalloproteinase inducer (EMMPRIN) were simultaneously measured in samples of each ADSC monoculture, BRCA monoculture, and ADSC-BRCA co-culture, in a human cytokine magnetic 30-plex (LHC6003M, Life technologies, Carlsbad, USA) and a human MMP magnetic Luminex Performance Assay (FCSTM07-7, LMPM000, LMPM972; R&D Systems, Minneapolis, USA) according to the manufacturers’ instructions. Conditioned media from six separate culture wells per culture condition were collected, pooled, and measured in six technical replicates. Samples were analyzed with a Luminex 200 instrument (BioRad). The median fluorescent intensity was determined and the cytokine/MMP concentration ascertained based on the standard curves for each cytokine/MMP. Prior to the analysis of the conditioned media of our human co- and monocultures we confirmed the specificity of the test for human proteins and excluded crossreactivity with bovine proteins, especially from FCS.

### Analysis of angiogenic properties

In order to determine the proangiogenic effect of ADSCs and BRCAs alone or in co-culture, supernatants of six separate culture wells of each condition were collected at day 5 of cell culture, pooled, and analyzed for induction of tube formation in six separate wells in a tube formation assay kit with human umbilical vein endothelial cells (HUVECs; Merck Millipore # ECM 625) according to the manufacturer’s instructions. In brief, wells of a 96-well plate were coated with an ECM matrix solution and HUVEC cells were resuspended in the different conditioned media from ADSCs, BRCAs, or ADSC-BRCA co-cultures; 7500 HUVEC cells in conditioned medium were seeded onto the matrix in each well and incubated for 9 h. Tube formation was visualized with a light microscope at a magnification × 20. A positive control was induced by phorbol 12-myristate 13-acetate (PMA; Abcam, Cambridge, UK, no. ab120297).

Three representative pictures of each culture (monoculture, co-culture) were microscopically analyzed in a blinded fashion by four independent examiners. Differences in the angiogenic properties between co-culture and the respective monocultures were ranged in three categories (increased = 1, equal = 0, decreased angiogenesis = –1) according to number, length, and sprouting of tubes, as well as formation of loops. Mean scores from 0.25 to 0.33 were determined as light change, scores from 0.34 to 0.66 were determined as medium change, and scores above 0.66 were stated as strong change. Scores below 0.25 were determined as no significant change.

### Statistical analysis

The results of cellular proliferation, migration, and invasion assays were evaluated by SigmaStat Software (SYSTAT) using student’s *t* test. *P* values <0.05 were stated as significantly different. If the normality test failed a Wilcoxon signed rank test was performed.

## Results

### Determination of stemness

The stemness of the applied ADSCs was determined according to the joint statement of the International Federation for Adipose Therapeutics and Science (IFATS) and the International Society for Cellular Therapy (ISCT) [14] by analysis of distinct surface markers in flow cytometry and analysis of adipogenic and osteogenic differentiation with oil red and alizarin red staining, respectively.

#### Flow cytometry

ADSCs were positive for CD13, CD29, CD44, CD63, CD73, CD90, CD105, and CD166. ADSCs were negative for CD11b, CD31, CD34, CD45, CD106, and CD235a (Additional file 1: Figure S1).

#### Differentiation

Adipogenic and osteogenic differentiation were induced to evaluate the multipotent differentiation potential. In all donors, adipogenically induced cells showed a remarkably higher oil red staining than noninduced control cells (Additional file 2: Figure S2a). Osteogenically differentiated ADSCs showed remarkably higher extracellular calcium deposition than noninduced control cells, analyzed with alizarin red stain (Additional file 2: Figure S2b).

### Proliferation

**Fig. 1:**
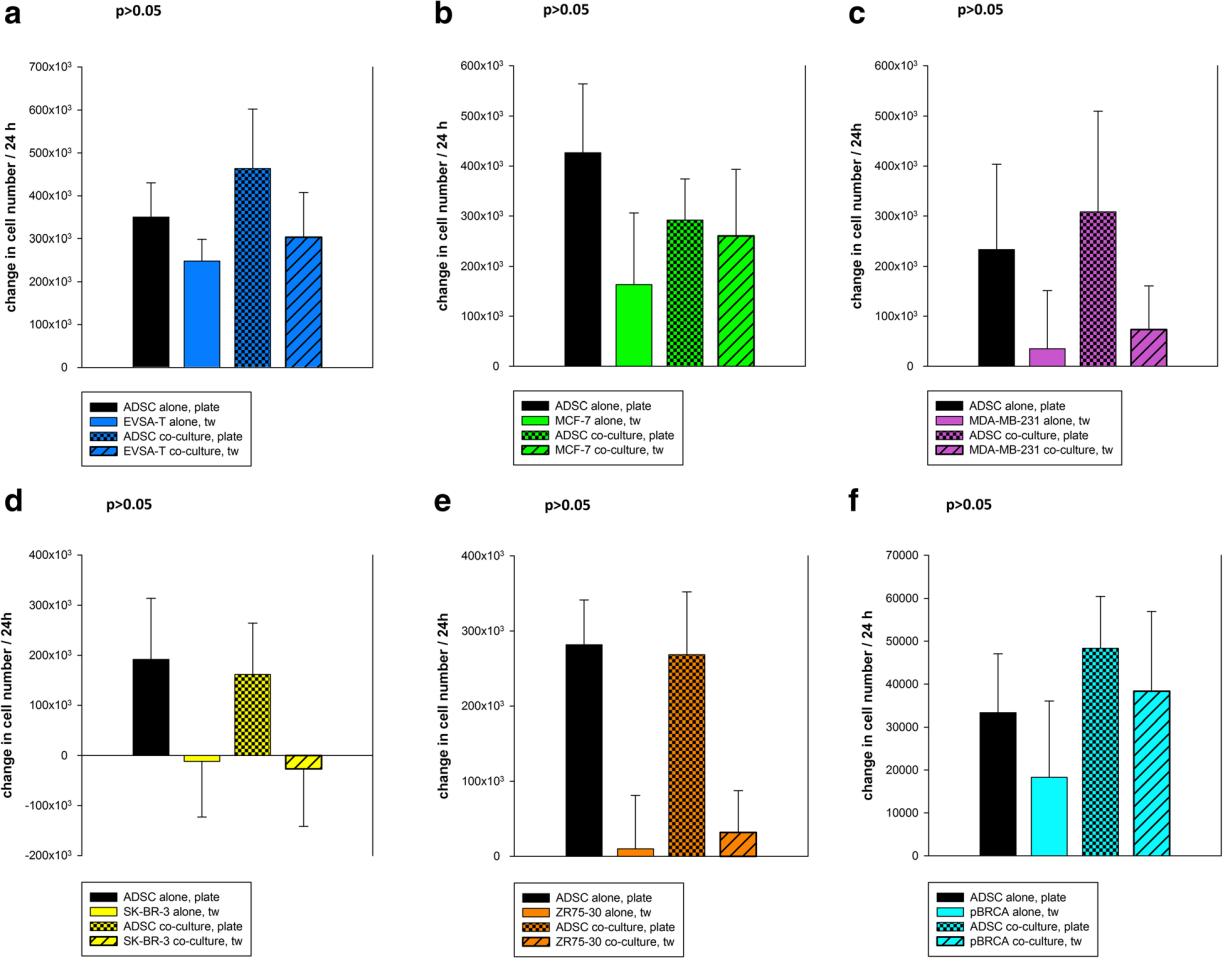
Effect of ADSC-BRCA co-culture on the proliferative activity of cells. **a** ADSC-EVSA-T co-culture: the growth of both cell types was not significantly affected (*p* > 0.05). **b** ADSC-MCF-7 co-culture: the growth of both cell types was not significantly affected (*p* > 0.05). **c** ADSC-MDA-MB-231 co-culture: the growth of both cell types was not significantly affected (*p* > 0.05). **d** ADSC-SK-BR-3 co-culture: the growth of both cell types was not significantly affected (*p* > 0.05). **e** ADSC-ZR75-30 co-culture: the growth of both cell types was not significantly affected (*p* > 0.05). **f** ADSC-pBRCA co-culture: the growth of both cell types was not significantly affected (*p* > 0.05). *ADSC* adipose-derived mesenchymal stem cell

Cell proliferation was determined by analyzing the doubling time of cells during the exponential growth phase (in general from day 2 to 4) (Fig. 1). Data are given as mean with standard deviation (SD).

#### ADSC-BRCA co-culture

Co-culture did not significantly affect cellular proliferation of either ADSCs or BRCAs or pBRCAs (*p* > 0.05).

### Quantitative real-time PCR

Co-cultured ADSCs and BRCAs showed distinct differences in the gene expression levels compared to a monoculture of ADSCs or BRCAs/pBRCAs (Table 1). To facilitate clear data presentation, only the results of the ADSCs on plates/BRCAs or pBRCAs in transwell inserts (co-culture), and changes in gene expression twofold or greater are displayed.

**Table 1:** Changes in the gene expression levels of ADSCs and BRCAs/pBRCAs in co-culture compared to monoculture

Gene name	Fold-change (monoculture/co-culture)	Up-/downregulation
Co-culture of ADSCs and EVSA-T		
Changes in the gene expression of ADSCs		
ETV4	2.3 (0.0)	↓
Co-culture of ADSCs and MCF-7		
Changes in the gene expression of ADSCs		
CCL-28	6.0 (0.3)	↑
CCNA2	3.0	↓
CCNB1	3.0	↓
CDC2/CDK1	3.5	↓
CDH-1	Undetermined → CT 36.1	↑
CX3CL-1	4.4 (1.1)	↑
CXCL-12	5.8 (0.2)	↑
E2F2	4.2	↓
GDF-5	2.6 (0.1)	↓
GPR81/HCAR1	2.1 (0.1)	↓
IL-6	2.3 (0.1)	↑
KDR	6.2	↓
MMP1	4.4 (0.1)	↓
TLR2	2.6 (0.6)	↓
TNFSF-10	3.0 (0.2)	↑
PPP1R1B	8.7	↑
PTGS2	3.6 (0.2)	↑
TUBA4A	3.0	↓
WISP-1	21 (1.8)	↑
Changes in the gene expression of MCF-7		
IL18	2.1 (0.1)	↓
KDR	3.0	↑
TNFSF-10	4.6 (0.5)	↑
Co-culture of ADSCs and MDA-MB-231		
Changes in the gene expression of ADSCs		
CCL-8	2.4 (0.1)	↑
CSF-3	8.3 (0.8)	↑
CXCL-1	10 (0.4)	↑
CXCL-2	2.3 (0.3)	↑
CXCL-3	3.2 (0.1)	↑
CXCL-6	9.7 (0.3)	↑
IL-6	3.0 (0.2)	↑
IL-8	4.6 (0.4)	↑
MMP1	2.6 (0.3)	↓
Changes in the gene expression of MDA-MB-231		
CXCL-2	3.7 (0.1)	↑
CXCL-3	4.2 (0.1)	↑
IL-6	2.3 (0.1)	↑
IL-8	4.7 (0.1)	↑
MMP-3	4.8 (0.2)	↑
MMP-10	7.2 (0.8)	↑
PSCA	3.5 (0.7)	↑
TLR-2	2.0 (0.3)	↑
Co-culture of ADSCs and SK-BR-3		
Changes in the gene expression of ADSCs		
CCL-20	2.4 (0.5)	↓
CXCL-1	3.5 (0.0)	↓
CSF-3	5.7 (0.4)	↓
CXCL-3	5.0 (0.1)	↓
CXCL-12	2.4 (0.0)	↑
IL-8	5.5 (0.2)	↓
PTGS2	3.2 (0.1)	↓
SERPINB5	2.4 (1.1)	↓
TLR2	2.6 (0.4)	↓
TNFSF-10	3.3 (0.1)	↑
Changes in the gene expression of SK-BR-3		
CCL-20	2.5 (0.0)	↑
CCL-26	3.0 (0.3)	↑
CEACAM-1	3.0 (0.3)	↑
CSF-3	2.4 (0.5)	↓
CXCL-1	2.6 (0.1)	↑
EPHB2	2.3 (0.2)	↑
MCAM	2.1 (0.1)	↑
MMP2	4.4 (0.1)	↑
SERPINB5	2.9 (0.7)	↓
RECK	2.0 (0.2)	↓
STAT4	7.7 (1.2)	↑
TLR-2	2.0 (0.3)	↑
TNFSF-10	2.2 (0.2)	↑
TMPRSS-4	2.7 (0.4)	↑
Co-culture of ADSCs and ZR75-30		
Changes in the gene expression of ADSCs		
CX3CL-1	4.6 (0.7)	↑
CXCL-10	3.0 (0.6)	↑
CXCL-14	2.0 (0.3)	↑
CXCR4	3.2 (0.2)	↓
MET	2.0 (0.1)	↓
PSCA	2.3 (0.1)	↓
Changes in the gene expression of ZR75-30		
CCL2	2.3 (0.1)	↑
FXYD5	3.0 (0.1)	↑
PSCA	7.9 (0.2)	↓
CXCL-14	7.4 (1.5)	↓
MET	4.4 (1.1)	↓
MMP-2	5.4 (1.9)	↓
STAT4	5.1 (0.7)	↑
Co-culture of ADSCs and primary BRCAs		
Changes in the gene expression of ADSCs		
CCL-7	7.5 (0.2)	↑
CD44	2.0 (0.0)	↓
CD82	10 (0.4)	↑
EPHB2	2.1 (0.1)	↑
FGF-2	3.9 (0.1)	↑
GNRH1	2.1 (0.2)	↓
KISS1R	8.6 (2.5)	↓
MMP-1	17 (0.8)	↑
MMP-3	13 (1.3)	↑
PSCA	4.3 (0.3)	↓
S100A4	2.0 (0.1)	↓
SERPINE1	8.0 (0.1)	↑
TNFSF-10	3.5 (0.2)	↓
WISP1	33 (0.7)	↑
Changes in the gene expression of primary BRCAs		
CD82	2.1 (0.2)	↑
DARC	2.8 (0.1)	↓
GNRH1	2.3 (0.1)	↑
MMP-1	2.3 (0.1)	↑
MMP-3	3.4 (0.3)	↑
MMP-7	5.1 (0.5)	↑
TIMP-4	7.6 (1.8)	↑
TNFSF-10	3.0 (0.3)	↓
TMPRSS4	Undetermined → CT 32.4	↑
TWIST1	2.0 (0.0)	↑
WISP1	29 (0.9)	↑

GUSB was used as referring housekeeping-gene. Only changes twofold or higher are displayed. Arrows mark an up- (↑) or downregulation (↓) of the gene expression compared to the referring monoculture. Values in parentheses indicate the respective standard deviation

*CT* cycle threshold

#### Co-culture of EVSA-T and ADSCs

In the co-cultured ADSCs, a change of the gene expression level could only be found in the expression of ETV4 (2.3-fold upregulation) compared to the respective monoculture. There was no remarkable change in the gene expression of the EVSA-T cells.

#### Co-culture of MCF-7 and ADSCs

In the co-cultured ADSCs, a clear increase of the gene expression level could be found in WISP-1 (21-fold), CCL-28 (6.0-fold), CXCL-12 (5.8-fold), CX3CL-1 (4.4-fold), PTGS2 (3.6-fold), TNFSF-10 (3.0-fold), IL-6 (2.3-fold), and E-Cadherin (CDH-1, from undetermined to CT 36.1) compared to the respective monoculture. A downregulation was determined for MMP-1 (4.4-fold), TLR2 and GDF-5 (2.6-fold), and GPR81/HCAR1 (2.1-fold).

When MCF-7 BRCAs were co-cultured with ADSCs the expression of TNFSF-10 (4.6-fold) was upregulated in the MCF-7 BRCAs, and the expression of IL-18 (2.1-fold) was downregulated compared to the respective monoculture.

#### Co-culture of MDA-MB-231 and ADSCs

In the co-cultured ADSCs, a clear increase of the gene expression level could be found in CXCL-1 (10-fold), CXCL-6 (9.7-fold), CSF-3 (8.3-fold), IL-8 (4.6-fold), CXCL-3 (3.2-fold), IL-6 (3.0-fold), CCL-8 (2.4-fold), and CXCL-2 (2.3-fold) compared to the respective monoculture. A downregulation was determined for MMP-1 (2.6-fold).

When MDA-MB-231 BRCAs were co-cultured with ADSCs the following genes were upregulated in the MDA-MB-231 BRCAs: MMP-10 (7.2-fold), MMP-3 (4.8-fold), IL-8 (4.7-fold), CXCL-3 (4.2-fold), CXCL-2 (3.7-fold), PSCA (3.5-fold), IL-6 (2.3-fold), and TLR-2 (2.0-fold). No genes analyzed were remarkably downregulated in co-cultured MDA-MB-231 BRCAs.

#### Co-culture of SK-BR-3 and ADSCs

In the co-cultured ADSCs, a clear increase of the gene expression level could be found in TNFSF10 (3.3-fold) and CXCL-12 (2.4-fold) compared to the respective monoculture. A downregulation was determined for CSF-3 (5.7-fold), IL-8 (5.5-fold), CXCL-3 (5.0-fold), CXCL-1 (3.5-fold), PTGS2 (3.2-fold), TLR2 (2.6-fold), SERPINB5 and CCL20 (2.4-fold).

When SK-BR-3 BRCAs were cocultured with ADSCs the following genes were upregulated in the SK-BR-3 BRCAs: STAT4 (7.7-fold), MMP-2 (4.4-fold), CCL26 and CEACAM-1 (3.0-fold), CXCL-1 (2.6-fold), CCL20 (2.5-fold), TMPRSS4 (2.7-fold), EPHB2 (2.3-fold), TNFSF10 (2.2-fold), MCAM (2.1-fold), and TLR-2 (2.0-fold). SERPINB5 (2.9-fold), CSF-3 (2.4-fold), and RECK (2.0-fold) were remarkably downregulated in co-cultured SK-BR-3 BRCAs.

#### Co-culture of ZR75-30 and ADSCs

In the co-cultured ADSCs, a clear increase of the gene expression level could be found in CX3CL-1 (4.6-fold), CXCL-10 (3.0-fold), and CXCL-14 (2.0-fold) compared to the respective monoculture. A downregulation was determined for CXCR4 (3.2-fold), PSCA (2.3-fold), and MET (2.0-fold).

When ZR75-30 BRCAs were co-cultured with ADSCs the following genes were upregulated in the ZR75-30 BRCAs: STAT4 (5.1-fold), FXYD5 (3.0-fold), and CCL2 (2.3-fold). PSCA (7.9-fold), CXCL-14 (7.4-fold), MMP-2 (5.4-fold), and MET (4.4-fold) were remarkably downregulated in co-cultured ZR75-30 BRCAs.

#### Co-culture of pBRCAs and ADSCs

In the co-cultured ADSCs, a strong increase of the gene expression level could be found for WISP-1 (33-fold), MMP-1 (17-fold), MMP-3 (13-fold), CD82 (10-fold), SERPINE1 (8.0-fold), and CCL-7 (7.5-fold), and a moderate increase for FGF-2 (3.9-fold) and EPHB2 (2.1-fold). A downregulation was determined for KISS1R (8.6-fold), PSCA (4.3-fold), TNFSF-10 (3.5-fold), GNRH1 (2.1-fold), CD44 and S100A4 (2.0-fold).

When pBRCAs were co-cultured with ADSCs the gene expression of WISP (29-fold), TIMP4 (7.6-fold), and MMP-7 (5.1-fold) was strongly upregulated. Furthermore, a moderate increase in the gene expression of MMP-3 (3.4-fold), GNRH1 (2.3-fold), MMP-1 (2.3-fold), CD82 (2.1-fold), TWIST1 (2.0-fold), and TMPRSS4 (from undetermined to CT 32.4) was detected. The expression of DARC (2.8-fold) and TNFSF-10 (3.0-fold) was downregulated on co-culture compared to the respective monoculture.

### Multiplex protein analysis (Table 2)

**Table 2:** Changes in the protein expression levels of ADSCs and BRCAs/pBRCAs in co-culture compared to monoculture

Protein	ADSC monoculture (pg/ml)	Respective BRCA monoculture (pg/ml)	Co-culture (pg/ml)	Fold-change for ADSC (monoculture/co-culture)	Fold-change for respective BRCA (monoculture/co-culture)
Co-culture of ADSCs and EVSA-T cell line					
CCL2	1755 (65)	ND	1421 (108)	0.9 →	NA ↑↑
EMMPRIN	594 (46)	208 (7.6)	828 (37)	1.4 →	4.0 ↑
HGF	1803 (159)	ND	1104 (155)	0.6 →	NA ↑↑
IL-6	62 (11)	ND	53 (7.1)	0.9 →	NA ↑↑
IL-7	8.4 (6.3)	ND	14 (6.8)	1.7 →	NA ↑
IL-8	4261 (673)	ND	3818 (665)	0.9 →	NA ↑↑
MMP-1	165,407 (32,918)	n.d	80,345 (3137)	0.5 →	NA ↑↑
MMP-2	61,271 (3320)	ND	66,795 (5514)	1.1 →	NA ↑↑
MMP-3	8274 (355)	23 (1.5)	5356 (142)	0.7 →	233 ↑↑
MMP-7	208 (40)	ND	156 (29)	0.8 →	NA ↑
MMP-9	82 (5.4)	32 (2.8)	70 (2.9)	0.9 →	2.2 ↑
MMP-10	385 (23)	21 (2.7)	258 (6.8)	0.7 →	12 ↑
VEGF	47 (0.0)	ND	38 (4.6)	0.8 →	NA ↑↑
Co-culture of ADSCs and MCF-7 cell line					
CCL2	2004 (99)	ND	1007 (75)	0.5 →	NA ↑↑
CCL3	16 (0.7)	14 (1.6)	17 (2.0)	1.1 →	1.2 →
CCL5	5.1 (3.4)	12 (7.6)	4.9 (2.6)	1.0 →	0.4 ↓
CXCL10	2.3 (0.1)	1.9 (0.1)	2.1 (0.2)	0.9 →	1.1 →
EMMRPIN	768 (34)	551 (28)	1073 (57)	1.4 →	2.0 →
Eotaxin	1.0 (0.1)	1.2 (0.2)	0.9 (0.1)	0.8 →	0.8 →
G-CSF	91 (9.7)	29 (5.2)	55 (8.3)	0.6 →	1.9 →
GM-CSF	1.7 (0.1)	1.0 (0.1)	1.3 (0.1)	0.7 →	1.3 →
HGF	2095 (244)	ND	1488 (67)	0.7 →	NA ↑↑
IL-4	18 (1.0)	12 (0.6)	16 (1.3)	0.9 →	1.3 →
IL-5	2.8 (0.1)	2.8 (0.2)	2.9 (0.3)	1.0 →	1.0 →
IL-6	160 (20)	ND	160 (23)	1.0 →	NA ↑↑
IL-8	5330 (633)	ND	2982 (367)	0.6 →	NA ↑↑
IL-10	4.6 (0.5)	4.0 (0.4)	4.9 (0.4)	1.1 →	1.2 →
IL-12	11.7 (4.2)	ND	10.6 (5.1)	0.9 →	NA ↑
IL-13	10 (1.0)	15 (0.5)	20 (1.7)	2.0 ↑	1.3 →
IL-1Ra	24 (1.7)	24 (1.1)	25 (1.5)	1.0 →	1.0 →
INF-α	80 (6.5)	ND	46 (7.3)	0.6 →	NA ↑↑
MMP-1	85,387 (18,267)	30 (5.4)	17,881 (1437)	0.2 →	596 ↑↑
MMP-2	51,127 (3291)	ND	74,585 (6060)	1.5 →	NA ↑↑
MMP-3	3514 (281)	40 (0.0)	3,905 (173)	1.1 →	98 ↑↑
MMP-9	112 (7,6)	112 (3.2)	99 (4.0)	0.9 →	0.9 →
MMP-10	193 (76)	41 (3.9)	236 (6.7)	1.2 →	5.8 ↑
VEGF	44 (4.7)	ND	48 (6.1)	1.1 →	NA ↑↑
Co-culture of ADSCs and MDA-MB-231 cell line					
CCL2	965 (140)	2.4 (0.0)	949 (162)	1.0 →	395 ↑↑
EMMPRIN	496 (20)	434 (30)	882 (53)	1.8 →	2.0 →
HGF	889 (206)	9.2 (4.9)	708 (129)	0.8 →	77 ↑↑
IL-4	1.7 (1.0)	1.4 (0.0)	0.9 (0.6)	0.5 →	0.6 →
IL-6	199 (32)	80 (3.9)	207 (36)	1.0 →	2.6 ↑
IL-7	22 (2.6)	17 (2.0)	15 (6.4)	0.7 →	0.9 →
IL-8	3238 (528)	350 (32)	2836 (474)	0.9 →	8.1 ↑
MMP-1	58,500 (5616)	5637 (414)	32,448 (3927)	0.6 →	5.8 ↑
MMP-2	17,767 (1796)	ND	18,290 (1907)	1.0 →	NA ↑↑
MMP-3	2185 (79)	196 (11)	1994 (118)	0.9 →	10 ↑
MMP-9	105 (4.0)	93 (6.1)	101 (5.8)	1.0 →	1.1 →
MMP-10	160 (7.0)	50 (3.1)	151 (11)	0.9 →	3.0 ↑
VEGF	22 (12)	24 (0.0)	25 (9.2)	1.1 →	1.0 →
Co-culture of ADSCs and SK-BR-3 cell line					
bFGF	4.8 (0.0)	ND	ND		
CCL2	1200 (134)	24 (5.8)	699 (92)	0.6 →	29 ↑↑
EMMPRIN	566 (22)	456 (13)	1030 (50)	1.8 →	2.3 →
HGF	975 (159)	ND	537 (94)	0.6 →	NA ↑↑
IL-4	1.2 (0.9)	ND	0.6 (0.6)	0.5 →	NA ↑↑
IL-6	175 (20)	4.3 (1.6)	145 (15)	0.8 →	34 ↑↑
IL-7	13 (8.1)	ND	6.9 (5.6)	0.5 ↓	NA ↑↑
IL-8	3581 (263)	66 (15)	2540 (337)	0.7 →	39 ↑↑
IL-1Ra	20 (0.0)	ND	ND	NA ↓	NA →
INF-α	30 (7.5)	ND	ND	NA ↓	NA →
MMP-1	62,844 (4604)	29 (2.0)	37,601 (2334)	0.6 →	1297 ↑
MMP-2	42,296 (2367)	ND	59,467 (4706)	1.4 →	NA ↑↑
MMP-3	3472 (316)	48 (3.0)	3261 (251)	0.9 →	68 ↑↑
MMP-9	111 (8.5)	255 (14)	128 (10)	1.2 →	0.5 →
MMP-10	231 (15)	33 (13)	219 (10)	1.0 →	6.4 ↑
VEGF	24 (9.7)	ND	23 (7.9)	1.0 →	NA ↑↑
Co-culture of ADSCs and ZR75-30 cell line					
CCL2	3603 (375)	112 (11)	3137 (212)	0.9 →	28 ↑↑
CCL3	21 (2.1)	16 (1.4)	18 (2.0)	0.9 →	1.2 →
CCL4	2.2 (0.7)	ND	2.7 (0.0)	1.2 →	NA ↑
CCL5	7.8 (4.1)	6.4 (2.2)	3.1 (1.2)	0.4 ↓	0.5 →
CXCL10	2.9 (0.3)	2.1 (0.1)	2.3 (0.3)	0.8 →	1.1 →
EMMPRIN	819 (40)	21 (2.5)	594 (4.9)	0.7 →	28 ↑↑
Eotaxin	1.2 (0.3)	1.3 (0.2)	0.9 (0.1)	0.8 →	0.7 →
G-CSF	85 (15)	32 (8.5)	54 (14)	0.6 →	1.7 →
GM-CSF	2.2 (0.4)	1.1 (0.0)	1.7 (0.2)	0.8 →	1.5 →
HGF	4672 (557)	ND	2589 (239)	0.6 →	NA ↑↑
IL-4	21 (3.0)	13 (0.0)	18 (1.6)	0.9 →	1.4 →
IL-5	3.0 (0.2)	2.8 (0.1)	3.0 (0.3)	1.0 →	1.1 →
IL-6	186 (42)	ND	151 (10)	0.8 →	NA ↑↑
IL-7	24 (11)	ND	ND	NA ↓↓	NA ↓↓
IL-8	4686 (714)	33 (7.8)	2578 (316)	0.6 →	78 ↑↑
IL-10	5.2 (0.3)	4.2 (0.3)	5.0 (0.8)	1.0 →	1.2 →
IL-12	20 (5.8)	ND	13 (8.5)	0.7 →	NA ↑↑
IL-13	23 (2.8)	16 (0.9)	20 (1.7)	0.9 →	1.3 →
IL-1Ra	28 (3.6)	26 (1.4)	26 (3.2)	0.9 →	1.0 →
INF-α	116 (9.7)	ND	90 (6.3)		
INF-γ	1.2 (0.8)	0.2 (0.2)	2.1 (0.0)	1.8 →	11 ↑
MMP-1	116,547 (31,612)	18 (0.6)	43,964 (2942)	0.4 ↓	2442 ↑↑
MMP-2	121,997 (6892)	ND	131,537 (2758)	1.1 →	NA ↑↑
MMP-3	5264 (444)	43 (2.0)	2665 (210)	0.5 →	62 ↑↑
MMP-9	110 (4.2)	89 (5.3)	106 (6.5)	1.0 →	1.2 →
MMP-10	292 (17)	39 (2.6)	173 (6.6)	0.6 →	4.4 ↑
VEGFA	56 (8.4)	ND	49 (5.0)		
Co-culture of ADSCs and primary BRCAs					
bFGF	8.7 (0.0)	60 (9.2)	46 (7.7)	5.3 ↑	0.8 →
CCL2	5241	24,396 (2324)	35,559 (1471)	16 ↑↑	1.4 →
CCL3	ND	135	126	NA ↑↑	1.0 →
CCL4	ND	37	48	NA ↑↑	1.3 →
CCL5	37 (11)	136 (9.2)	110 (14)	3.0 ↑	0.8 →
CXCL-9	ND	271 (9.2)	553 (68)	20 ↑	2.0 →
CXCL-10	6.5 (0.4)	572 (9.2)	89 (15)	13.6 ↑	6.4 ↓
EGF	ND	71 (9.2)	49 (22)	NA ↑↑	0.7 →
EMMRPIN	104 (12)	1908 (281)	1949 (122)	19 ↑↑	1.0 →
Eotaxin	1.1 (0.6)	4.2 (9.2)	6.5 (1.4)	5.8 ↑	1.6 →
G-CSF	ND	1604 (9.2)	1396 (142)	NA ↑	0.8 →
GM-CSF	6.8 (0.3)	23 (9.2)	37 (0.7)	5.4 ↑	1.5 →
HGF	1740 (99)	398 (9.2)	1254 (99)	0.7 →	3.2 ↑
IL-1b	ND	84 (9.2)	75 (22)	NA →	0.9 →
IL-2	ND	10 (9.2)	7.3 (2.6)	NA →	0.7 →
IL-4	40 (4.2)	105 (9.2)	121 (6.3)	3.0 ↑	1.2 →
IL-5	ND	5.1 (9.2)	6.9 (1.7)	NA ↑	1.3 →
IL-6	1222 (114)	7768 (1007)	12,121 (409)	20 ↑↑	1.5 →
IL-7	45 (37)	248 (9.2)	251 (32)	5.6 ↑	1.0 →
IL-8	3224 (150)	17,925 (2899)	34,099 (1586)	11 ↑↑	1.9 →
IL-10	22 (1.7)	62 (9.2)	56 (4.6)	2.6 ↑	0.9 →
IL-12	47 (5.2)	179 (9.2)	207 (11)	4.4 ↑	1.2 →
IL-13	23 (4.1)	57 (9.2)	63 (11)	2.7 ↑	1.1 →
IL-15	48 (22)	363 (9.2)	235 (49)	4.9 ↑	0.7 →
IL-17	ND	22	27	NA ↑	1.2 →
IL-2R	90 (65)	398 (9.2)	425 (113)	4.7 ↑	1.0 →
INF-α	172 (16)	950 (9.2)	1186 (35)	6.9 ↑	1.3 →
INF-y	4.7 (0.0)	24 (9.2)	20 (6.4)	4.4 ↑	0.8 →
MMP-1	8045 (483)	3606 (509)	12,850 (1750)	1.6 →	3.6 ↑
MMP-2	75,480 (3780)	17,192 (2131)	108,563 (4932)	1.4 →	6.3 ↑
MMP-3	1218 (51)	757 (145)	5005 (395)	4.1 ↑	6.6 ↑
MMP-9	94 (7.4)	873 (107)	852 (50)	9.0 ↑	1.0 →
MMP-10	82 (2.5)	542 (70)	857 (54)	11 ↑↑	1.6 →
TNF-α	2.0 (2.6)	18 (9.2)	20 (4.2)	9.8 ↑	1.1 →
VEGF	131 (16)	518 (9.2)	629 (32)	4.8 ↑	1.2 →

Standard deviation is given in brackets

Results from 0 to 9.9 are shown with one decimal point, results for 10 or higher are displayed without decimal points

*ND* not detectable, *NA* not applicable

#### Co-culture of EVSA-T and ADSCs

In co-culture with ADSCs, EVSA-T BRCAs experienced a strong increase in the protein levels of MMP-3 (233-fold), and MMP-10 (12-fold), and a moderate increase in EMMPRIN (4.0-fold), and MMP-9 (2.2-fold). Exclusively in co-culture, EVSA-T BRCAs are exposed to CCL2, HGF, IL-6, -7, -8, MMP-1, -2, -7, and VEGF.

Co-cultured ADSCs were not exposed to major differences in protein levels compared to the respective monoculture.

#### Co-culture of MCF-7 and ADSCs

In co-culture with ADSCs, MCF-7 BRCAs experienced a strong increase in the protein concentration of MMP-1, MMP-3, and MMP-10 (5.8–596-fold changes). Exclusively in co-culture, MCF-7 BRCAs were exposed to CCL-2, HGF, IL-6, IL-8, IL-12, INF-α, MMP-2, and VEGF. ADSCs were not exposed to major changes in the protein levels in co-culture compared to monoculture (0.4–2.0-fold changes).

#### Co-culture of MDA-MB-231 and ADSCs

In co-culture with ADSCs, MDA-MB-231 BRCAs experienced a strong increase in the protein concentration of CCL2, IL-8, HGF, MMP-1, and MMP-3, and a mild increase in IL-6 and MMP-10 (2.6–395-fold changes). Exclusively in co-culture, MDA-MB-231 BRCAs were exposed to MMP-2. ADSCs were not exposed to major changes in the protein levels in co-culture compared to monoculture (0.5–1.8-fold changes).

#### Co-culture of SK-BR-3-BRCAs and ADSCs

In co-culture with ADSCs, SK-BR-3 BRCAs experienced a strong increase in the protein concentration of CCL2, IL-6, IL-8, MMP-1, MMP-3, and MMP-10 (6.4–1297-fold changes). Exclusively in co-culture, SK-BR-3 BRCAs were exposed to HGF, IL-7, MMP-2, and VEGF. ADSCs were not exposed to major changes in the protein levels in co-culture compared to monoculture (0.5–1.8-fold changes).

#### Co-culture of ZR75-30 and ADSCs

In co-culture with ADSCs, ZR75-30 BRCAs experienced a strong increase in the protein concentration of CCL2, EMMPRIN, IL-8, MMP-1, and MMP-3, as well as a moderate increase in the protein level of MMP-10 (4.4– 2442-fold changes). Exclusively in co-culture, ZR-75-30 BRCAs were exposed to CCL4, HGF, IL-6, MMP-2, and IL-12. ADSCs were not exposed to major changes in the protein levels in co-culture compared to monoculture (0.4–1.2-fold changes).

#### Co-culture of pBRCA and ADSCs

In co-culture with ADSCs, primary BRCAs experienced a strong increase in the protein concentration of MMP-2 and MMP-3 (6.3- and 6.6-fold) and a moderate increase in the level of MMP-1 and HGF (3.6- and 3.2-fold). A strong decrease was measured in the concentration of CXCL-10 in co-culture compared to pBRCA monoculture (6.4-fold).

ADSCs co-cultured with pBRCAs experienced a strong increase in the protein concentration of IL-6, EMMPRIN, CCL2, CXCL-10, MMP-10, TNF-α, MMP-9, INF-α, Eotaxin, IL-7, GM-CSF, bFGF, IL-15, VEGF, IL-2R, IL-12, INF-γ, and MMP-3 (4.1–20-fold). A moderate increase was seen in the levels of CCL5, IL-4, IL-13, and IL-10 (2.6–3.0-fold).

Exclusively in co-culture, ADSCs were exposed to CCL-3, CCL-4, CXCL-9, G-CSF, IL-1b, IL-2, IL-5, and IL-17.

### Migration

**Fig. 2:**
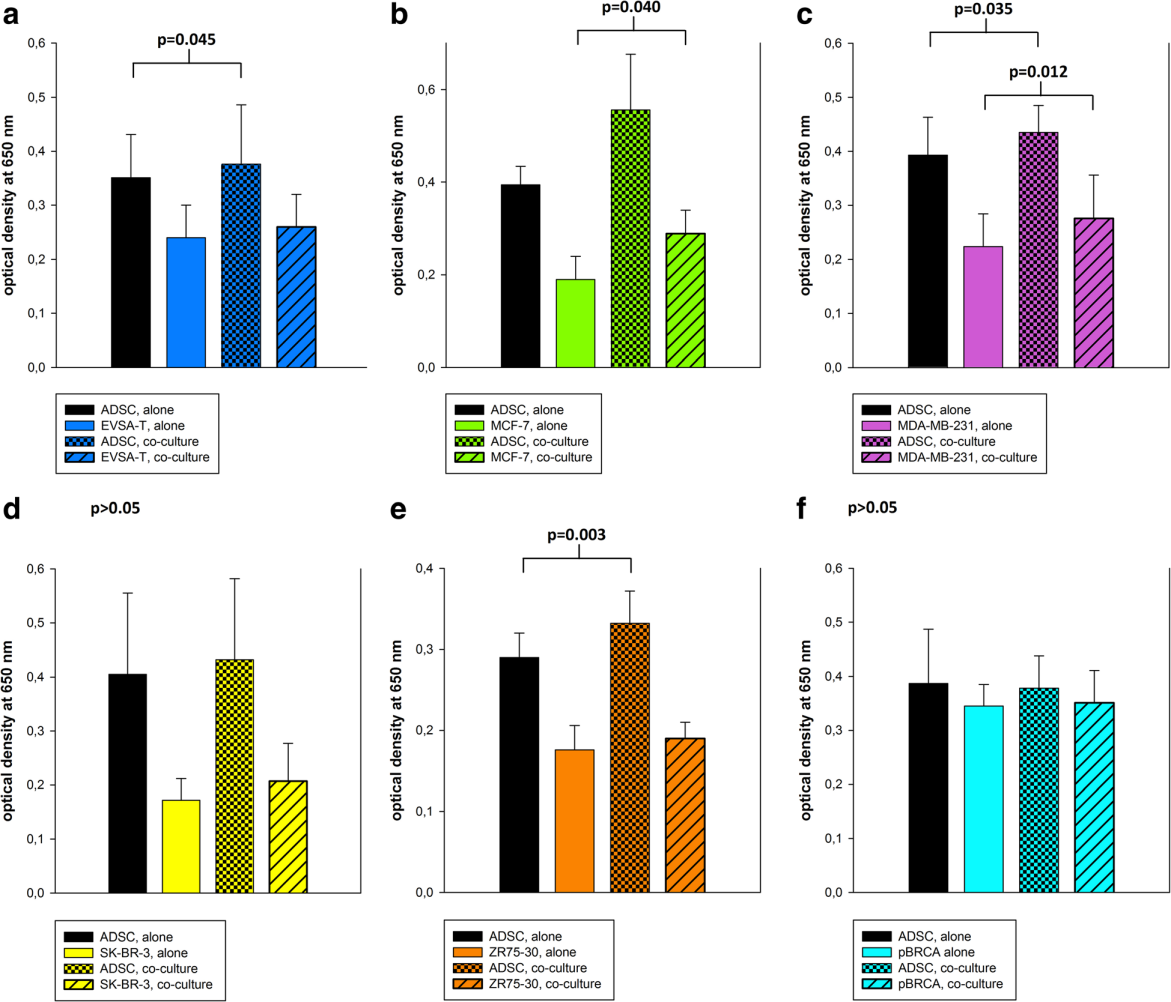
Migration capacity of ASDCs and BRCAs alone and in co-culture. The migratory capacity was measured as optical density at 650 nm. **a** ADSC-EVSA-T co-culture: co-cultured ADSCs showed a significantly higher migration than in monoculture (*p* = 0.045). There was no significant change in the migratory behavior of EVSA-T BRCAs in co-culture. **b** ADSC-MCF-7 co-culture: co-cultured MCF-7-BRCAs showed a significantly higher migration than in monoculture (*p* = 0.040). There was no significant change in the migratory behavior of ADSCs in co-culture. **c** ADSC-MDA-MB-231 co-culture: co-cultured ADSCs (*p* = 0.035) and co-cultured MDA-MB-231 (*p* = 0.012) showed a significantly higher migration than in monoculture. **d** ADSC-SK-BR-3 co-culture: there was no significant change in the migratory behavior of ADSCs or SK-BR-3 BRCAs in co-culture compared to the respective monoculture (*p* > 0.05). **e** ADSC-ZR75-30 co-culture: co-cultured ADSCs showed a significantly higher migration than in monoculture (*p* = 0.003). There was no significant change in the migratory behavior of ZR75-30 BRCA in co-culture. **f** ADSC-pBRCA co-culture: there was no significant change in the migratory behavior of ADSCs or pBRCAs in co-culture compared to the respective monoculture (*p* > 0.05). Values are shown with standard deviations. *ADSC* adipose-derived mesenchymal stem cell

The migration through the transwell pores could already be detected when ADSCs or the different BRCAs were cultured alone; however, when co-cultured, the migration of MCF-7 BRCAs was significantly increased about 11% (*p* = 0.04) compared to monoculture. MDA-MB-231 BRCAs also showed significantly higher migration (+23%) in co-culture with ADSCs (*p* = 0.012). Furthermore, ADSCs co-cultured with MDA-MB-231, ZR-75-30, or EVSA-T BRCAs showed significantly higher migration (+11%, +15%, and +12%, respectively) than ADSCs in monoculture (*p* = 0.035, 0.003, and 0.045, respectively). There were no significant differences in the migration of EVSA-T, SK-BR-3, ZR-75-30, or pBRCAs in co-culture compared to monoculture (*p* > 0.05) (Fig. 2).

### Invasion

**Fig. 3:**
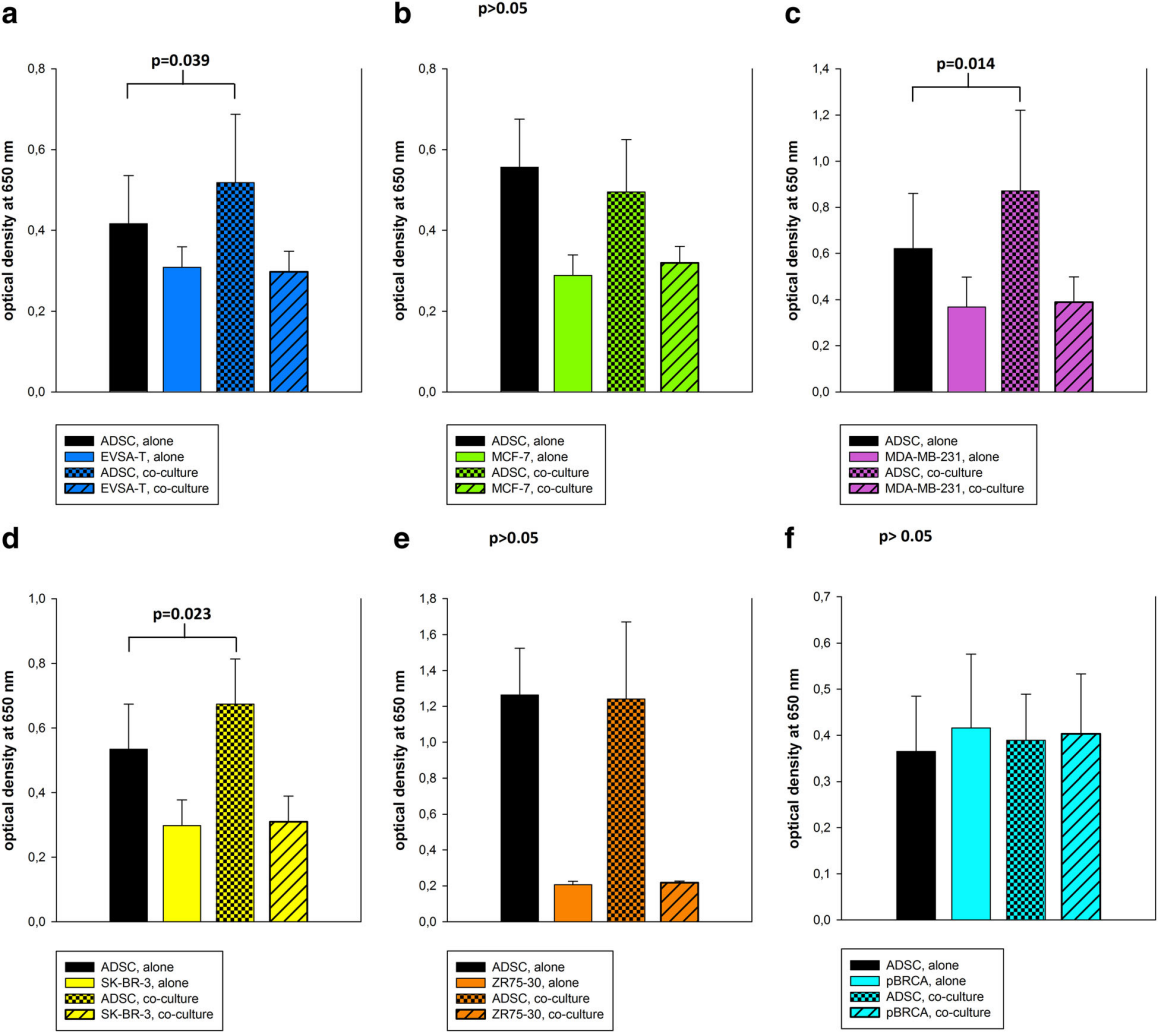
Invasion assay of ASDCs and BRCAs alone and in co-culture. Invasive capacity was measured as optical density at 650 nm. **a** ADSC-EVSA-T co-culture: co-cultured ADSCs showed a significantly higher invasive activity than in monoculture (*p* = 0.039). There was no significant change in EVSA-T BRCA invasive activity in co-culture. **b** ADSC-MCF-7 co-culture: there was no significant change in ADSC or MCF-7 invasive activity in co-culture compared to the respective monoculture (*p* > 0.05). **c** ADSC-MDA-MB-231 co-culture: co-cultured ADSCs showed a significantly higher invasive activity than in monoculture (*p* = 0.039). There was no significant change in MDA-MB-231 BRCA invasive behavior in co-culture. **d** ADSC-SK-BR-3 co-culture: co-cultured ADSCs showed a significantly higher invasive activity than in monoculture (*p* = 0.023). There was no significant change in SK-BR-3-BRCA invasive behavior in co-culture. **e** ADSC-ZR75-30 co-culture: there was no significant change in ADSC or ZR75-30 invasive activity in co-culture compared to the respective monoculture (*p* > 0.05). **f** ADSC-pBRCA co-culture: there was no significant change in ADSC or pBRCA invasive behavior in co-culture compared to the respective monoculture (*p* > 0.05). Values are shown with standard deviations. *ADSC* adipose-derived mesenchymal stem cell

Both ADSCs and the different BRCA cell lines showed invasive behavior by actively digesting the extracellular matrix blocking the transwell pores and migrating to the lower surface of the floor of the transwell inserts. In co-culture with MDA-MB-231, SK-BR-3, or EVSA-T cells, ADSCs showed significantly higher invasive properties (41%, 26%, and 24%, respectively) compared to ADSCs from monoculture (*p* = 0.014, 0.023, and 0.039, respectively). There were no significant differences in the invasive properties of MCF-7, MDA-MB-231, SK-BR-3, ZR-75-30, EVSA-T, and pBRCAs in co-culture compared to monoculture (*p* > 0.05) (Fig. 3).

### Angiogenesis

**Fig. 4:**
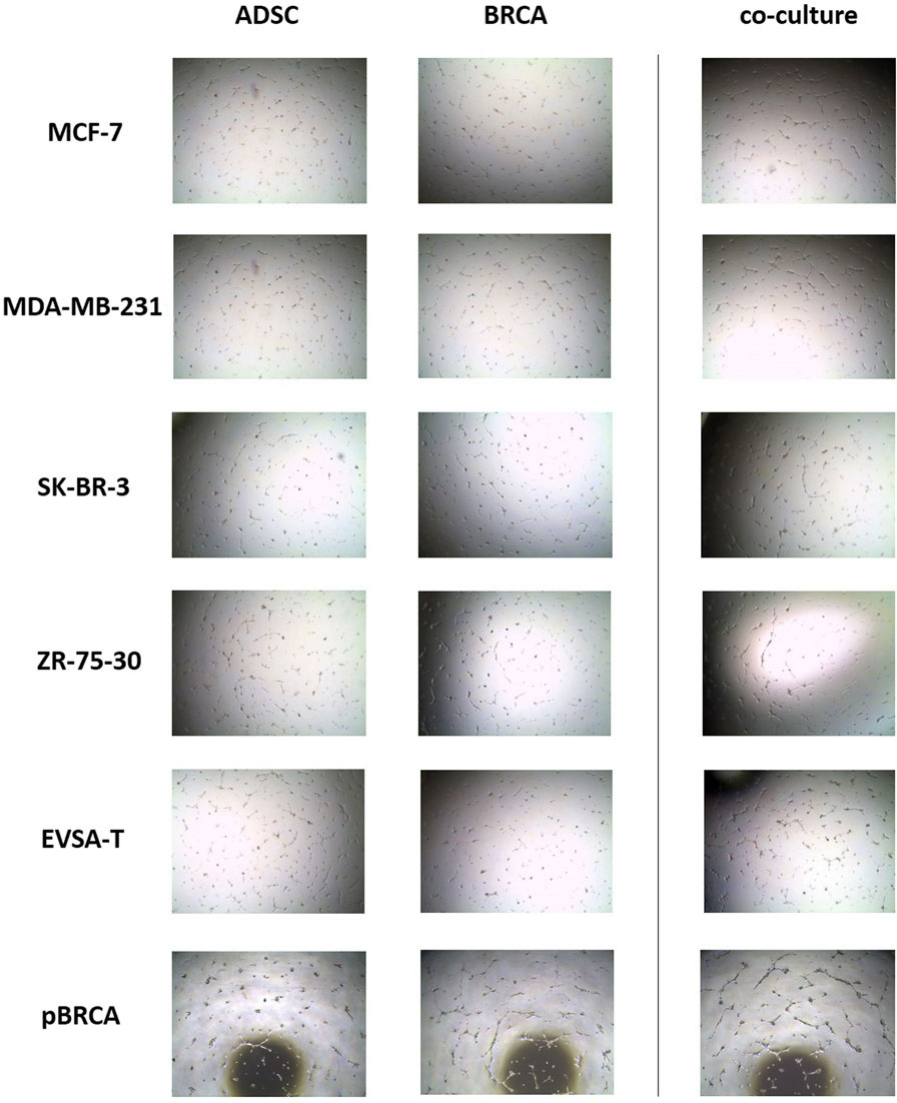
Induction of angiogenesis. Representative pictures of tube formation assay wells for all conditions (magnification × 20). Tube formation could be detected after incubation of HUVECs with conditioned media from co-cultured ADSCs-BRCAs as well as from ADSC and BRCA (primary and cell lines) monoculture. The co-culture of ADSCs and BRCAs led to an increase in angiogenic properties in both ADSCs and BRCAs for all cell lines. For ADSCs, a strong increase in angiogenesis could be seen in co-culture with MCF-7 and primary BRCAs, a moderate increase in co-culture with MDA-MB-231, and a slight increase in co-culture with SK-BR-3 and ZR-75-30. Co-culture with EVSA-T did not change the induction of angiogenesis. For BRCAs, a strong increase in the angiogenic properties in co-culture with ADSCs was found for MCF-7, MDA-MB-231, and SK-BR-3 cells, a moderate increase for ZR-75-30, and slight increase for EVSA-T. We could not detect a significant influence of co-culture on primary BRCAs. *ADSC* adipose-derived mesenchymal stem cell

Tube formation could be detected after incubation of HUVECs with conditioned media from co-cultured ADSCs-BRCAs as well as from ADSC and BRCA (primary and cell lines) monocultures. The co-culture of ADSCs and BRCAs lead to an increase in angiogenic properties in both ADSCs and BRCAs for all cell lines. For ADSCs, a strong increase in angiogenesis could be seen in co-culture with MCF-7 and primary BRCAs, a moderate increase in co-culture with MDA-MB-231, and a slight increase in co-culture with SK-BR-3 and ZR-75-30. Co-culture with EVSA-T did not change the induction of angiogenesis. For BRCAs, a strong increase in the angiogenic properties in co-culture with ADSCs was found for MCF-7, MDA-MB-231, and SK-BR-3 cells, a moderate increase for ZR-75-30, and a slight increase for EVSA-T. We could not detect a significant influence of co-culture on primary BRCAs (Fig. 4).

## Discussion

ADSCs and BRCAs secrete a variety of messenger proteins linked to malignancy-associated properties in their microenvironment. In vitro co-culture and presumably also co-localization in vivo exposes the respective cell types to new proteins or a changed concentration of, for example, cytokines, growth factors, and metalloproteinases.

The most important change for BRCAs co-cultured with ADSCs in our experiment was a tremendous increase in the concentration of CCL2, HGF, IL-6 and -8, and MMP-1, -2, -3, and -10. Interestingly, ADSCs co-cultured with BRCA cell lines did not experience a significant change in the analyzed protein levels; when co-cultured with primary BRCAs, however, they were also exposed to significantly increased levels of a range of cytokines, growth factors, and proteinases secreted by the tumor cells. This could either be due to the fact that the tested cancer cell lines might have lost some important features of cellular interactions with ADSCs throughout their process of immortalization or be a consequence of the different media conditions of cell lines and primary cells. To further elucidate and confirm these different effects we are currently analyzing more primary cancer cells.

In our current experiment, we determined a robust upregulation of different C-C motif ligand-chemokines (CCL), such as CCL2, -7, -20, -26, and -28, and C-X-C motif ligand-chemokines (CXCL), such as CXCL1, -2, -3, -6, -10, -12, and -14 on the gene expression level in co-culture in both cell types. Some of them are well known for supporting promalignant features, such as tumor cell proliferation, migration, invasion, or angiogenesis. They promote tumor growth and facilitate metastasis, and high serum levels have been associated with a poor prognosis [15–19]. CCL2 has been suggested to promote cancer cell survival, regulate CAF-carcinoma cell interactions and fuel late-stage carcinoma progression. It is known as a major chemotactic factor secreted by BRCAs, CAFs, and a special leukocyte/monocyte subpopulation, tumor-associated macrophages (TAMs), and has been shown to significantly increase the migration of ADSCs towards the tumor or metastatic site in a dose-dependent manner [7, 20]. Our data show that ADSCs are a robust source of CCL2 secretion in monoculture and also in co-culture with BRCAs. BRCA cell lines only produce minor amounts of CCL2 while, in contrast, the primary BRCA population secretes an extraordinarily high amount of CCL2. In our experiment, ADSCs led to a strong increase in the overall CCL2 levels in co-culture with both primary BRCAs and BRCA cell lines. In line with this, we found a significantly increased migration of co-cultured ADSCs towards EVSA-T, MDA-MB-231, and ZR75-30 BRCAs. Furthermore, in vitro co-culture with ADSCs also significantly increased the migration of MCF-7 and MDA-MB-231 BRCAs, which is consistent with previous results [10]. In tumors, chemokines such as CCL2, CCL5, IL-8, and CXCL-12 also chemoattract TAMs to the tumor site. There, high numbers of TAMs are correlated with poor prognosis and disease progression [21]. TAMs produce a multitude of tumor-promoting growth factors, as well as MMPs and urokinase-type plasminogen activator. They promote tumor growth and metastasis by stimulating angiogenesis, ECM degradation, and tumor cell proliferation, and inhibit the antitumor immune response [6]. CCL2 and CCL5 were also shown to stimulate the secretion of proteases by TAMs. Thus, by secreting remarkable amounts of CCL2 and CCL5, ADSCs that have been recruited into the tumor stroma could promote tumor progression by further increasing the number of TAMs in the tumor microenvironment.

Only primary BRCAs and, at very low levels, MDA-MB-231 secreted HGF in our experiment. In co-culture with ADSCs, however, all BRCAs were exposed to very high levels of HGF, due to the HGF secretion of ADSCs. As a stroma-derived factor, HGF has been shown to be involved in cancer progression, especially in EMT, invasion, and metastasis [6, 22]. Rowan et al. found ADSCs to increase micrometastasis in an in vivo MDA-MB-231 mouse tumor model [20]. In our experiment, HGF secretion by ADSCs was high, but not further increased in co-culture. We could not detect a significant increase in the in vitro invasive properties of the different tumor cell lines or the primary BRCAs in co-culture, despite the significantly higher HGF concentration. ADSCs, however, showed significantly increased invasive capacities in co-culture with EVSA-T, MDA-MB-231, and SK-BR-3. This might in part be a result of the increased MMP secretion in co-culture. ADSCs secrete high levels of various MMPs, such as MMP-1, -2, -3, -9, and -10. Interestingly, this has also been shown for CAFs in the tumor microenvironment [6]. MMPs not only degrade extracellular matrix and thereby enable invasion, they also facilitate neoangiogenesis and thus further support tumor growth. Altered MMP expression has already been linked to poor disease prognosis in different human cancers and enhanced cancer cell invasion [23, 24]. In our study, MMP-2 showed an absolute increase in its protein concentration in the conditioned medium of all BRCA-ADSC co-cultures, above the summarized levels of the respective monocultures. This fits with the recent finding that MMP-2 in breast cancer metastasis is predominantly expressed in the tumor stroma, and that MMP-2 plays an important role in breast cancer tumor growth, progression, and metastasis [25]. Furthermore, stromal MMP-2 expression has been closely associated with different clinicopathologic parameters and overall survival of breast cancer patients [26]. Thus, co-localization of ADSCs and BRCAs might increase BRCA malignant progression in vivo by increasing the overall level of MMP-2.

We also determined a significantly upregulated WISP-1 gene expression (21–33-fold) in co-cultured primary BRCAs and ADSCs, as well as in ADSCs co-cultured with MCF-7. The role of WISP-1 gene expression in breast cancer and its prognostic value has been discussed controversially [27, 28]. The WISP-1 gene encodes the Wnt-induced secreted protein-1, which is associated with the extracellular matrix and interacts with cellular integrins. It has been shown to enhance tumor cell migration by increasing MMP-2 expression, to inhibit antitumor immunity by blocking the response of immune cells to IL-12, and to act antiapoptotically by inducing or inhibiting different signaling pathways [29–31]. In line with this, we found an absolute increase in MMP-2 expression in all co-cultures, and a significantly increased migration in co-cultured MCF-7 and MDA-MB-231 BRCAs. The upregulated WISP-1 gene expression in co-localized BRCAs and ADSCs—as found in our study—could lead to a poorer prognosis in vivo, as has already been shown in different tumor types [29, 30, 32].

Additionally, ADSCs and primary BRCAs secreted remarkable amounts of IL-6 and IL-8, while all BRCA cell lines either do not express IL-6 or IL-8, or only in minor amounts. Co-culture with ADSCs thus exposes BRCAs to very high or much higher amounts of IL-6/IL-8 compared to the respective monoculture. IL-6 has been reported to be a proliferative factor for diverse tumor types in vivo [33–35]. Elevated serum levels of IL-6 have been associated with key features of malignancy, cancer progression, and a poor clinical outcome in different types of cancers [31–37]. IL-6 has furthermore been demonstrated on the leading edge of human breast cancer specimens in vivo and has been shown to correlate positively with advanced tumor stage. Secretion of IL-6 by BRCAs can also induce IL-6 expression in the surrounding cells, presumably by an IL-6/STAT3 autocrine/paracrine feed-forward loop. Through a JAK-dependent signaling cascade, IL-6 induces the phosphorylation of STAT3-tyrosine. This seems to be a major reason for the induction of metastasis, and the attraction of tumor-associated suppressive myeloid cells, endothelial, and stromal cells into the tumor, seen with increasing IL-6 levels in human breast cancer [36]. Thus, increasing the overall levels of IL-6 in the tumor microenvironment by adding ADSCs might have promalignant consequences in vivo.

IL-8 mediates its biological effects through two cell-surface G protein-coupled receptors, termed CXCR1 and CXCR2, found on cancers cells, endothelial cells, neutrophils, and TAMs. IL-8 binding to the receptor leads to the regulation of the activity of multiple transcription factors, modulates protein translation, and affects the organization of the cellular cytoskeleton by posttranslational regulation. IL-8 has been shown to support proliferation and survival/chemoresistance of cancer cells, to induce the infiltration of the tumor by neutrophils, and to stimulate the release of growth factors by TAMs. Furthermore, by stimulating endothelial cell proliferation, survival, chemotaxis, and migration, IL-8 stimulates angiogenesis and thereby supplies nutrition, an important component of tumor growth [37]. High IL-8 levels have been associated with a more advanced tumor stage, an accelerated clinical course with reduced postrelapse survival, and increased lymph node involvement in breast cancer patients [38]. In line with this, it has been shown previously that co-injection of MDA-MB231 and ADSCs leads to significantly higher tumor volumes and increased vessel densities compared to the injection of MDA-MB-231 alone, presumably through higher IL-8 and VEGF levels [10, 12, 20]. We could not detect significant effects of BRCA-ADSC co-culture on cellular proliferation in co-cultures of ADSCs or BRCAs. However, we found an increased angiogenesis in all co-cultured BRCAs and almost all ADSC co-cultures, and an upregulated IL-8 gene expression in MDA-MB-231-ADSC co-cultures. In addition, co-culture of ADSCs and primary BRCAs showed a strong total increase in IL-8 protein concentration, and a corresponding induction of angiogenesis. Accordingly, ADSCs could also promote tumor growth in vivo by secreting IL-8 and thereby supporting angiogenesis.

We also found a robust upregulation of TIMP-4 gene expression in co-cultured primary BRCAs. The TIMP-4 gene encodes for the tissue inhibitor of metalloproteinase-4. It inhibits BRCA apoptosis in vitro and in vivo and has also been linked to protumorigenic effects [39]. Increased levels of TIMP-4 in BRCA tumors have been associated with tumor progression of ductal carcinoma in situ, and a shorter disease-free survival in BRCA patients, especially in estrogen receptor (ER)-negative tumors [40].

TNFSF10, encoding for TRAIL (TNF-related apoptosis-inducing ligand), is significantly downregulated in co-culture of pBRCAs and ADSCs in both cell types. TRAIL binding to its receptors, TRAIL-R1 and TRAIL-R2, can lead to apoptosis by activation of caspase-8 and a downstream stimulation of caspase-3/7. Furthermore, TRAIL seems to be linked to the antitumor immune response mediated by natural killer cells and IFN-γ [41]. Decreased TRAIL activity has been associated with increased tumor growth and metastasis. In contrast to our results of the primary BRCAs, we found TNFSF-10 to be remarkably upregulated in tumor and stem cells in co-cultures of ADSCs with SK-BR-3 and MCF-7. This might be explained by the fact that many BRCA cell lines, such as SK-BR-3 and MCF-7, are resistant to TRAIL-induced apoptosis, closely connected with their hormonal receptor status. Triple-negative cell lines, such as MDA-MB-231, seem to be more sensitive than HER-2/neu or ER-positive cell lines. TRAIL sensitivity and actions are influenced by multiple factors, such as changes in the expression of interfering genes and receptors, regulating proteins, or medication [42–44]. Thus, there is no simple answer to the consequences of TNFSF10 up- or downregulation. Importantly, there are also multiple alternative pathways of cellular apoptosis.

Additionally, gene expression of DARC (decoy chemokine receptor) was downregulated in primary BRCAs co-cultured with ADSCs (2.8-fold). DARC is known to have antitumorigenic effects, such as decreasing tumor growth, angiogenesis, and metastasis, and increasing necrosis, by binding and sequestering protumorigenic, and especially proangiogenic chemokines. Furthermore, it is thought to decrease CCL2 and MMP-9 expression levels. Accordingly, low levels of DARC have been associated with poor prognosis in breast cancer [45].

To summarize, the in vitro co-culture of ADSCs and breast cancers cells leads to considerable changes in multiple key parameters of malignancy. This points towards a potentially increased oncological risk in vivo, which should not be neglected when considering a clinical use of cell-assisted lipoaspirates in breast cancer patients.

## Conclusions

In this study we focused on the secretome of ADSCs and BRCAs, and how the indirect co-localization of both impacts their behavior. With this, we were able to analyze the respective changes in cellular proliferation, gene expression, migration, and invasion separately for each cell type. Our results show that ADSCs significantly affect multiple malignant features of BRCAs in vitro, such as gene expression, protein secretion, migration, and angiogenesis.

Thereby, ADSCs may strongly increase the risk of breast cancer tumor growth and metastasis in vivo if administered to the vicinity of premalignant or malignant mammary cells.

However, it is important to say that in vitro results can only give a hint towards the more complex situation in vivo; e.g., an in vitro system cannot show the important antitumor immune response and its inhibition by TAMs. Furthermore, the impact of direct cell-to-cell contact also needs to be elucidated. Thus, to get the necessary further insight, additional in vitro and vivo experiments need to be performed.

Nonetheless, already our current results need to fuel a necessary discussion about the safety of ADSC-based therapies, especially in the breast. Informed consent of patients to such procedures will need to include an increased risk of developing breast cancer, having a higher risk of relapse or a faster growth and dissemination of a preexisting breast cancer. Additionally, it seems crucial to rigorously screen all patients for premalignant or residual lesions prior to the injection of fat, stem cell-augmented fat, or isolated ADSCs in the breast or adjacent tissues, to avoid a potential co-localization of ADSCs and BRCAs.

## Additional files

Additional file 1: Figure S1. Flow cytometry of pooled ADSCs from donors 1 to 6. Black lines show isotype controls, red lines show pooled ADSCs. ADSCs were positive for CD13, CD29, CD44, CD63, CD73, CD90, CD105, and CD166. ADSCs were negative for CD11b, CD31, CD34, CD45, CD106, and CD235a. (JPG 155 kb)

Additional file 2: Figure S2. Representative light microscope pictures of adipogenically and osteogenically differentiated ADSCs. Magnification × 10. (a) Intracellular lipid droplets stained by oil red method as a marker of adipogenic differentiation on day 14 of differentiation. (b) Extracellular calcium deposition stained with alizarin red as a marker of osteogenic differentiation on day 14 of differentiation. Undifferentiated controls are not shown. (JPG 385 kb)

## Abbreviations

ADSC: Adipose tissue-derived mesenchymal stem cell
BMSC: Bone marrow-derived mesenchymal stem cell
BRCA: Human breast cancer cell line
BSA: Bovine serum albumin
CAF: Cancer-associated fibroblast
CAL: Cell-assisted lipotransfer
CCL: C-C motif ligand-chemokine
CT: Cycle threshold
CXCL: C-X-C motif ligand-chemokine
DMEM: Dulbecco’s modified Eagle’s medium
EGF: Epidermal growth factor
EMT: Epithelial-to-mesenchymal transition
ER: Estrogen receptor
FCS: Fetal calf serum
FGF: Fibroblast growth factor
G-CSF: Granulocyte colony-stimulating factor
GM-CSF: Granulocyte-macrophage colony-stimulating factor
HGF: Hepatocyte growth factor
HUVEC: Human umbilical vein endothelial cell
IFN: Interferon
IL: Interleukin
ITS: Insulin, transferrin, selenous acid
MMP: Matrix metalloproteinase
pBRCA: Primary human breast cancer cell
PBS: Phosphate-buffered saline
PCR: Polymerase chain reaction
SD: Standard deviation
TAM: Tumor-associated macrophage
TNF: Tumor necrosis factor
TRAIL: TNF-related apoptosis-inducing ligand
VEGF: Vascular endothelial growth factor
