# Supplementary figures and images for: The impact of human adipose tissue-derived stem cells on breast cancer cells: implications for cell-assisted lipotransfers in breast reconstruction
Source: Stem Cell Res Ther. 2017 May 25;8:121. doi: 10.1186/s13287-017-0579-1 (PMC5445287; doi:10.1186/s13287-017-0579-1)

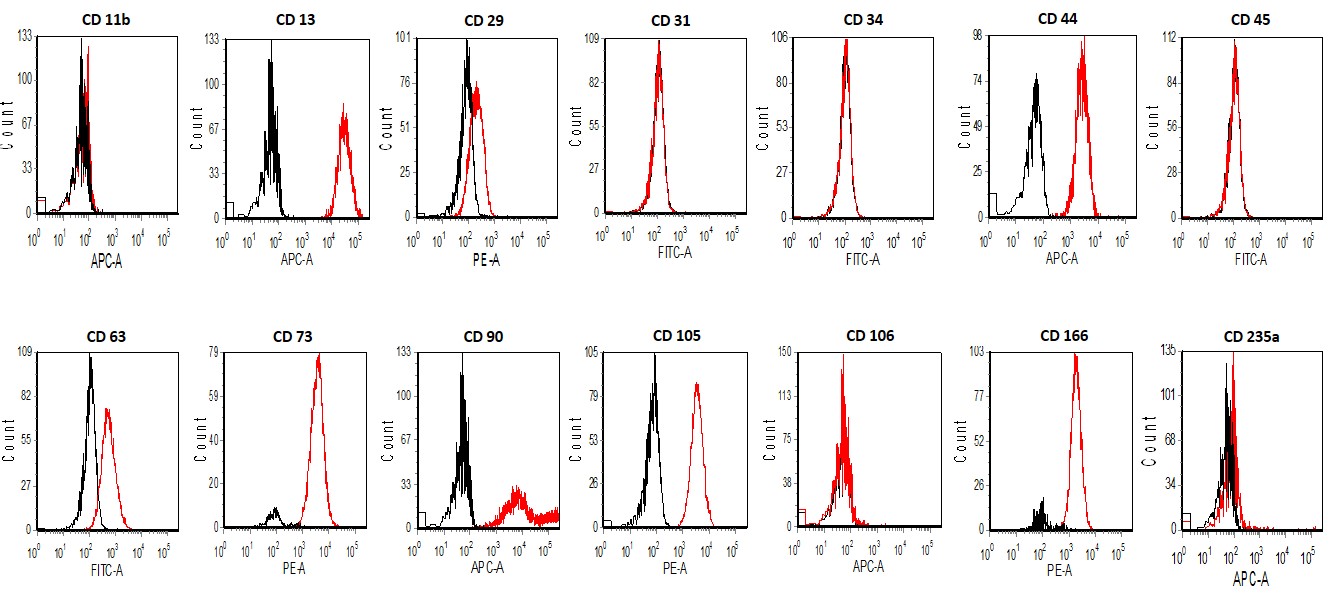

Supplement: Supplementary file 1 — Flow cytometry of pooled ADSCs from donors 1 to 6. Black lines show isotype controls, red lines show pooled ADSCs. ADSCs were positive for CD13, CD29, CD44, CD63, CD73, CD90, CD105, and CD166. ADSCs were negative for CD11b, CD31, CD34, CD45, CD106, and CD235a. (JPG 155 kb) [file 13287_2017_579_MOESM1_ESM.jpg]

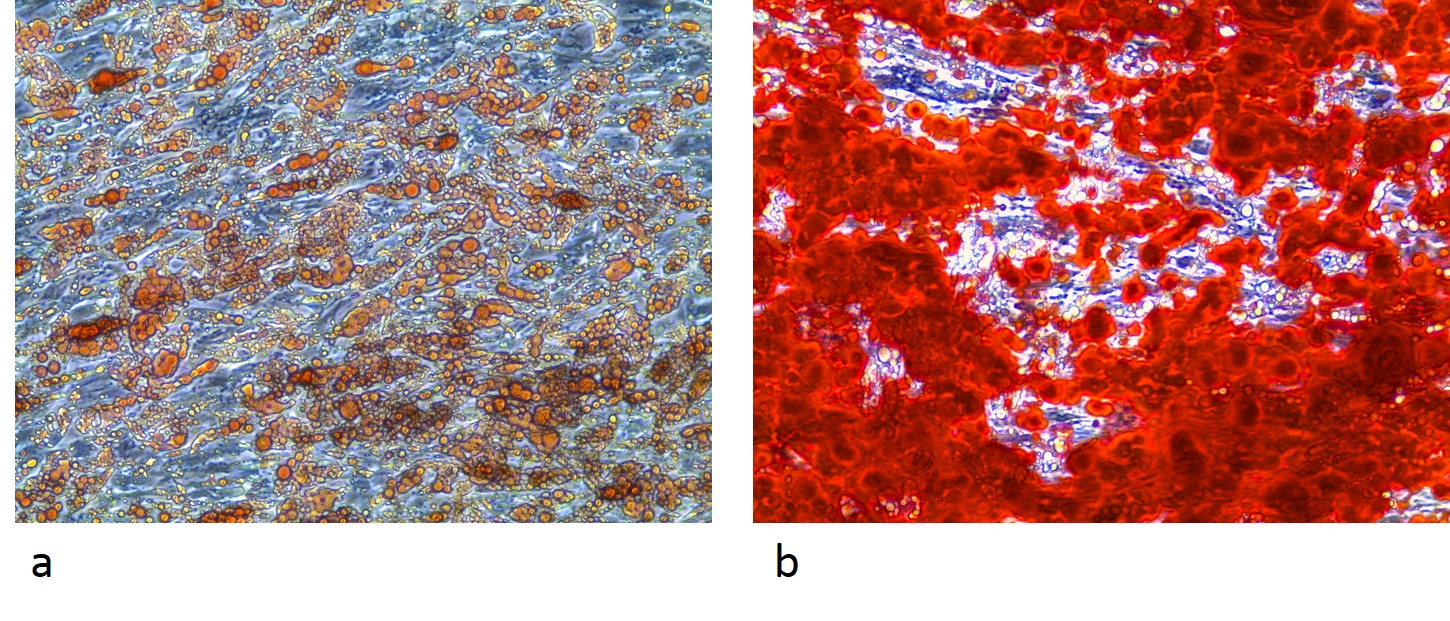

Supplement: Supplementary file 2 — Representative light microscope pictures of adipogenically and osteogenically differentiated ADSCs. Magnification × 10. (a) Intracellular lipid droplets stained by oil red method as a marker of adipogenic differentiation on day 14 of differentiation. (b) Extracellular calcium deposition stained with alizarin red as a marker of osteogenic differentiation on day 14 of differentiation. Undifferentiated controls are not shown. (JPG 385 kb) [file 13287_2017_579_MOESM2_ESM.jpg]
